# Acoustic behavior of melon-headed whales varies on a diel cycle

**DOI:** 10.1007/s00265-015-1967-0

**Published:** 2015-07-25

**Authors:** Simone Baumann-Pickering, Marie A. Roch, Sean M. Wiggins, Hans-Ulrich Schnitzler, John A. Hildebrand

**Affiliations:** Scripps Institution of Oceanography, University of California, San Diego, 9500 Gilman Dr. #0205, La Jolla, CA 92093 USA; Department of Computer Science, San Diego State University, 5500 Campanile Drive, San Diego, CA 92182-7720 USA; Animal Physiology, Institute for Neurobiology, University of Tübingen, Auf der Morgenstelle 28, 72076 Tübingen, Germany

**Keywords:** Adaptive acoustic behavior, Echolocation, Ambient noise, Melon-headed whale, Diel pattern

## Abstract

Many terrestrial and marine species have a diel activity pattern, and their acoustic signaling follows their current behavioral state. Whistles and echolocation clicks on long-term recordings produced by melon-headed whales (*Peponocephala electra*) at Palmyra Atoll indicated that these signals were used selectively during different phases of the day, strengthening the idea of nighttime foraging and daytime resting with afternoon socializing for this species. Spectral features of their echolocation clicks changed from day to night, shifting the median center frequency up. Additionally, click received levels increased with increasing ambient noise during both day and night. Ambient noise over a wide frequency band was on average higher at night. The diel adjustment of click features might be a reaction to acoustic masking caused by these nighttime sounds. Similar adaptations have been documented for numerous taxa in response to noise. Or it could be, unrelated, an increase in biosonar source levels and with it a shift in center frequency to enhance detection distances during foraging at night. Call modifications in intensity, directionality, frequency, and duration according to echolocation task are well established for bats. This finding indicates that melon-headed whales have flexibility in their acoustic behavior, and they collectively and repeatedly adapt their signals from day- to nighttime circumstances.

**Electronic supplementary material:**

The online version of this article (doi:10.1007/s00265-015-1967-0) contains supplementary material, which is available to authorized users.

## Introduction

Many organisms, from invertebrates to vertebrates, both in marine and terrestrial environments, have diel behavioral activity patterns, often observed with corresponding changes in the acoustic behavior. This has been documented, to name a few, for the dawn chorus of birds, nighttime echolocation of bats, nighttime calls of moths, crepuscular chorus of fish, and dusk calling of frogs (Griffin 1958; Kamimura and Tatsuki 1993; Staicer et al. 1996; Bridges and Dorcas 2000; Širović et al. 2009). A number of cetacean species show diel behavior that may be regulated by an internal circadian clock or may be linked to daily cycles in prey behavior (Klinowska 1986). Diel acoustic behavior has been shown in some baleen whale species using autonomous acoustic monitoring techniques (Au et al. 2000; Wiggins et al. 2005; Oleson et al. 2007; Munger et al. 2008; Matthews et al. 2014). Traditionally, descriptions of diel behavior of many dolphin species were based on daylight visual observations, and nighttime foraging was inferred from evening foraging activity or stomach content analysis (Würsig and Würsig 1979; Norris and Dohl 1980; Amano et al. 1998). Toothed whale echolocation clicks, which generally are used to detect, characterize, and localize a target for spatial orientation or feeding (Au 1993), have been shown to increase in number and repetition rates at night for harbor porpoise (*Phocoena phocoena*), spinner dolphins (*Stenella longirostris*), and Risso’s dolphins (*Grampus griseus*) (Norris et al. 1994; Carlström 2005; Todd et al. 2009; Soldevilla et al. 2010a). Also, spinner dolphin acoustic activity typically is low in the morning hours during resting and increased whistling occurs during socializing in the afternoon (Norris et al. 1994). Two different Pacific white-sided dolphin (*Lagenorhynchus obliquidens)* click types, possibly from two distinct populations with different foraging strategies, exhibit diel patterns with one type being dominant during daytime hours and the other at night (Soldevilla et al. 2010b).

Most acoustically active species are capable of collectively adapting their signals to optimize communication dependent on their behavior and environment (Bradbury and Vehrencamp 1998). For example, bird populations have been found to adapt song modulation rates in forest versus open-country environments to overcome propagation challenges (Krebs and Davies 1993). Adaptations in the acoustic behavior are often associated with changes in the environment; for example, many echolocating bats shorten their signal duration and increase their bandwidth as they navigate closer to vegetation (e.g., Schaub and Schnitzler 2007). Adaptive acoustic behavior to changes in the soundscape has been a more recent focus, particularly in the context of anthropogenic noise and its impact (e.g., Brumm 2013). Soundscapes are shaped by geophonies (e.g., wind, waves, volcanic eruptions, earthquakes), biophonies (e.g., chorus of birds, stridulation of insects, spawning chorus of fish), and anthropophonies (e.g., vehicle motor noise, seismic surveys, explosions) (Farina 2014). With increased sounds, an acoustic signal may be masked, evoking compensating modifications in the acoustic properties of signals. Most commonly, animals increase their signal amplitude to improve the signal-to-noise ratio and therefore detectability. Initially observed and described for humans as the Lombard response (Lombard 1911), it has since been found in many terrestrial species (e.g., frogs, primates, bats, and songbirds; Sinnott et al. 1975; Schmidt and Joermann 1986; Cynx et al. 1998; Brumm and Todt 2002; Pytte et al. 2003; Brumm 2004; Penna et al. 2005; Tressler and Smotherman 2009). Beyond an increase in amplitude, other signal changes include extending duration (e.g., Penna et al. 2005; Bermudez-Cuamatzin et al. 2011), increasing repetition rate (e.g., Potvin et al. 2011), shifting the dominant frequency to a band unaffected by the noise (e.g., Lopez et al. 1988; Feng et al. 2006; Bermudez-Cuamatzin et al. 2011; Potvin et al. 2011), or a combination of these (e.g., Lopez et al. 1988; Tressler and Smotherman 2009).

Changes in the acoustic behavior in response to noise have also been shown for cetacean species. North Atlantic right whales (*Eubalaena glacialis*), killer whales (*Orcinus orca*), and beluga whales (*Delphinapterus leucas*) adjust the call structure of their tonal signals and pulsed-tone calls in response to natural or anthropogenic noise. Right whales increase their calling amplitude with increasing ambient noise (Parks et al. 2011). In the presence of anthropogenic noise, beluga whales may reduce their calling rate, change the occurrence of specific calls, increase their dominant call frequency (Lesage et al. 1999), and increase the signal amplitude (Scheifele et al. 2005). Killer whales have been shown to respond to vessel noise with an increase in call amplitude (Holt et al. 2009, 2011) and an increase in call duration (Wieland et al. 2010). Low frequency wind noise resulted in an upward shift in call frequency of offshore killer whales (Foote and Nystuen 2008).

Few studies of captive animals have shown adaptations of echolocation clicks in response to noise. A captive beluga whale increased the source level and peak frequency of its echolocation clicks when moved from the quieter San Diego Bay to the noisier Kaneohe Bay, Hawaii (Au et al. 1985). Au (1993) concluded from findings in two studies (Thomas et al. 1988; Thomas and Turl 1990) that false killer whales (*Pseudorca crassidens*) also shift their frequencies and increase their call amplitudes when confronted with a noisy environment. However, findings in studies with captive animals may not be reflective of what can be expected in field studies when animals may be operating at the limits of their sound production system. In contrast, Cuvier’s beaked whales reduce their overall echolocation click rates in the presence of ship noise (Aguilar Soto et al. 2006).

Melon-headed whales (*Peponocephala electra*) are regularly observed around Palmyra Atoll. They are pelagic dolphins that occur worldwide in tropical and subtropical oceanic waters (40° N–35° S) (Jefferson et al. 2008; Perryman 2009). They are mostly observed offshore in deep water and are a highly social species with 100–500 animals (up to 2000) per group (Jefferson et al. 2008). Melon-headed whales use daytime hours for resting and socializing, and feed during the night on mesopelagic prey (Brownell Jr. et al. 2009). Whistles of melon-headed whales are described as relatively simple up and down sweeps, as well as sinusoidal signals (Watkins et al. 1997; Frankel and Yin 2010). Echolocation clicks of melon-headed whales recorded during daytime hours have been shown to have species-specific properties allowing them to be classified (Baumann-Pickering et al. 2010).

In this study, we investigated the diel acoustic activity pattern of melon-headed whales from a year-long survey and the animals’ acoustic adaptations in correlation with changes in ambient sound. We tested the hypothesis that melon-headed whale acoustic activity changed with their diel behavioral pattern, comparable to that of spinner dolphins given their shared preferred prey and similar behavioral diel pattern.

## Materials and methods

### Data collection

**Fig. 1:**
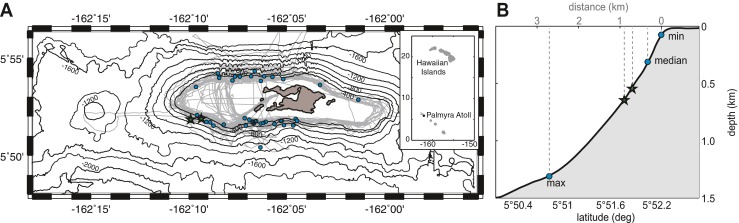
**a** Bathymetric map of Palmyra Atoll, with positions of HARPs (*stars*), visual survey tracklines (*gray*), and melon-headed whale sightings (*blue circles*). *Top right inset* shows approximate location of Palmyra Atoll in relation to the Hawaiian Islands. **b** Schematic approximation of the slope near the HARP locations (*stars*). Depth of melon-headed whale daytime sighting locations (minimum, median, maximum) is indicated with *circles*

An autonomous high-frequency acoustic recording package (HARP) was placed on the steep slope off Palmyra Atoll’s western terrace to investigate cetacean acoustic behavior. The HARP design differed from what was described in Wiggins and Hildebrand (2007) as it was in a mooring configuration with the hydrophone floating 20 m above the seafloor. It recorded from October 19, 2006 until March 23, 2007 and from April 9, 2007 until September 18, 2007. The recording gap of 16 days between the two deployments corresponded to servicing of batteries and hard drives. During the first deployment, the HARP was located at 5° 51.85′ N, 162° 09.91′ W, and 650 m deep. It was then deployed about 1 km east of the initial location at 5° 51.88′ N, 162° 09.36′ W, and 550 m deep (Fig. 1). The recorder was set to a sampling frequency of 200 kHz with 16-bit quantization and scheduled with a recording sequence of 5 continuous minutes every 20 min. The HARP used an omni-directional transducer (ITC-1042, International Transducer Corporation, Santa Barbara, CA), which had an approximately flat (±2 dB) hydrophone sensitivity from 10 to 100 kHz of −200 dB re V/μPa. It was connected to a custom-built preamplifier board with band-pass filter. The preamplifiers were designed to flatten the frequency response of the ambient ocean noise, which provided greater gain at higher frequencies where ambient noise levels are lower and sound attenuation is higher (Wiggins and Hildebrand 2007). The calibrated system response was corrected for during analysis.

### Signal processing

**Fig. 2:**
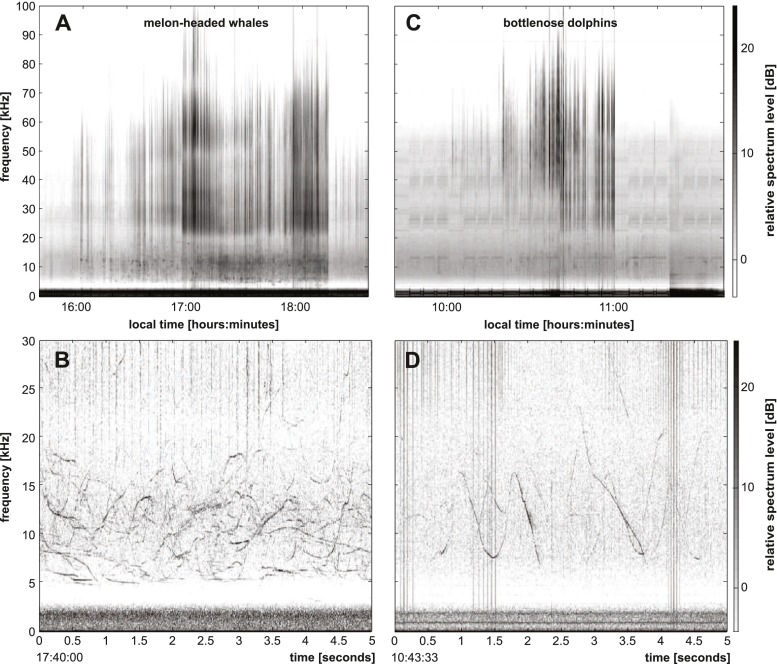
Example whistles and clicks on long-term recordings at Palmyra Atoll. **a**, **b** Signals from melon-headed whales, and **c**, **d** signals from bottlenose dolphins. *Top*: long-term spectral average (LTSA) showing several hours of recording. 2000-points FFT, 5 s average, 0 % overlap. *Bottom*: spectrogram showing 5 s of recording. 3000-points FFT, 80 % overlap. Graphs are not adjusted for system response

Signal processing was performed using the MATLAB (Mathworks, Natick, MA) based custom software program *Triton* (Wiggins and Hildebrand 2007) and other custom MATLAB routines. Melon-headed whale whistles in the HARP data were identified manually by a trained analyst (SBP). Long-term spectral averages (LTSAs, Wiggins and Hildebrand 2007) were calculated for visual analysis of the long-term recordings. LTSAs are time compressed spectrograms created using the Welch algorithm (Welch 1967). Each 5-s time bin of the LTSA consists of the average of 500 non-overlapped Hann-windowed spectra. The bins of averaged spectra were then aligned over time resulting in long-term spectrograms with a temporo-spectral resolution of 5 s × 100 Hz. The averaging process preserved short duration spectral features that would be lost in the Fourier transform of a 5-s window. The year’s data were manually analyzed through LTSAs (Fig. 2a, c). When whistles were notable in the LTSA, the sequence was inspected more closely with spectrograms typically of 5-s lengths, 3000-point DFTs, 80 % overlap, Hann window, and a frequency range of 0–30 kHz (Fig. 2b, d). Start and end times of these sequences were noted if the whistles were manually classified to originate from melon-headed whales, which were substantially different from whistles of other dolphins frequently encountered at Palmyra Atoll (e.g., bottlenose dolphins (*Tursiops truncatus*), Fig. 2c, 2d). These manual analyst decisions were based on differences in the signals, with melon-headed whale whistles being lower in frequency, shorter in duration, with less modulation than those of other delphinids commonly encountered in these waters (see “Supplementary Material 1”: Fig. S1, Table S1, and Table S2, as well as values from literature (Watkins et al. 1997; Frankel and Yin 2010)). To classify echolocation clicks as originating from melon-headed whales, we relied on the assumption that in a segment with whistles from this species the co-occurring clicks were produced by the same species. Only those echolocation sequences were used in further analysis that had several distinct whistles allowing for a confident analyst decision on the origin of these signals. However, occasional mixed species recordings within segments classified to have melon-headed whale signals cannot be ruled out.

Echolocation clicks were automatically detected within the HARP long-term data using a two-step approach as described in Soldevilla et al. (2008). During the first step, clicks were detected using a frequency energy detector. Spectral frames (1024 FFT, 50 % overlap, Hann window) were assumed to contain echolocation clicks when 12.5 % of the frequency bins between 15 and 85 kHz exceeded a threshold of 10 dB. Individual echolocation clicks were identified within these regions using a Teager energy operator (Kaiser 1990; Kandia and Stylianou 2006) applied to the time-series waveform as described in Roch et al. (2011). The recording sequences with detected clicks were digitally filtered with a 10-pole Butterworth band-pass filter. The low frequency cutoff was set to 5 kHz to minimize the influence of low frequency noise from boats and weather. The high-frequency cutoff was set to 95 kHz. Filtering was done on 800 samples. Spectra of each detected signal were calculated using 2.56 ms of data and a 512-point Hann window centered on the click. A calibrated transfer function was applied to account for the frequency dependent instrument response. Peak frequency was extracted as the frequency with the highest level within each click spectrum. Click received levels were computed from waveform peak-to-peak signal amplitudes and adjusted for the system response at center frequency of each click. The HARP raw data format used 75-s segments, making this a convenient analysis length. To ensure statistical independence of data points, only the first 75-s segment per 20 min was used in further analysis. All clicks within each segment with peak-to-peak received levels less than 130 dB re 1 μPa were discarded to allow only strong clicks well above the noise level in both nighttime as well as daytime situations. Segments with less than 20 clicks detected within a 75-s interval were not included into the analysis. To reduce variability in click data and to find species-specific features, medians over all click features of each 75-s segment were computed. To produce a dataset comparable to the published daytime data (Baumann-Pickering et al. 2010), a second set of medians per segment was calculated omitting clicks with peak frequency less than 20 kHz.

### Diel analysis

Sunrise and sunset data for Palmyra Atoll were acquired from the U.S. Naval Observatory website (http://aa.usno.navy.mil/data/docs/RS_OneYear.php). Sunrise occurred between 06:33 and 07:06, sunset between 18:29 and 19:01. These differences in sunrise and sunset were not considered substantial, so full hours of the day were pooled in the diel analysis. Hours between 07:00 and 19:00 were defined as daytime. Presence and absence data were calculated for 75-s HARP recording segments to count segments with whistles in each hour of the day using all manually identified acoustic encounters, presented as a histogram. Numbers of clicks per 75-s segment in each hour of the day were extracted from the reduced number of independent segments and presented as boxplot distributions. Two sample *t* tests with unequal variance were used to evaluate whether or not differences in measurements of click characteristics varied between day and night time echolocation click medians. The validity of the equal variance condition was evaluated through their F-ratios.

For the calculation of ambient noise levels, each 75-s segment within the first 30 min of each hour was, like the click data, filtered between 5 and 95 kHz with a 10-pole Butterworth band-pass filter. Root mean square (RMS) levels were calculated for the quietest segment of each hour when melon-headed whale whistles were present. Median RMS levels were calculated to compare daytime versus nighttime noise levels. The relationship of click peak-to-peak received levels and ambient noise RMS received levels was tested with a linear regression for day- and nighttime segments separately. Noise spectra were computed over the quietest segment using a 2048-point DFT and the Welch algorithm (Welch 1967) with no overlap over one exemplary week. Spectral levels were extracted for each hour at 5, 10, 30, and 50 kHz. Spectral levels of each of these frequencies were normalized to the lowest value within each frequency.

## Results

**Fig. 3:**
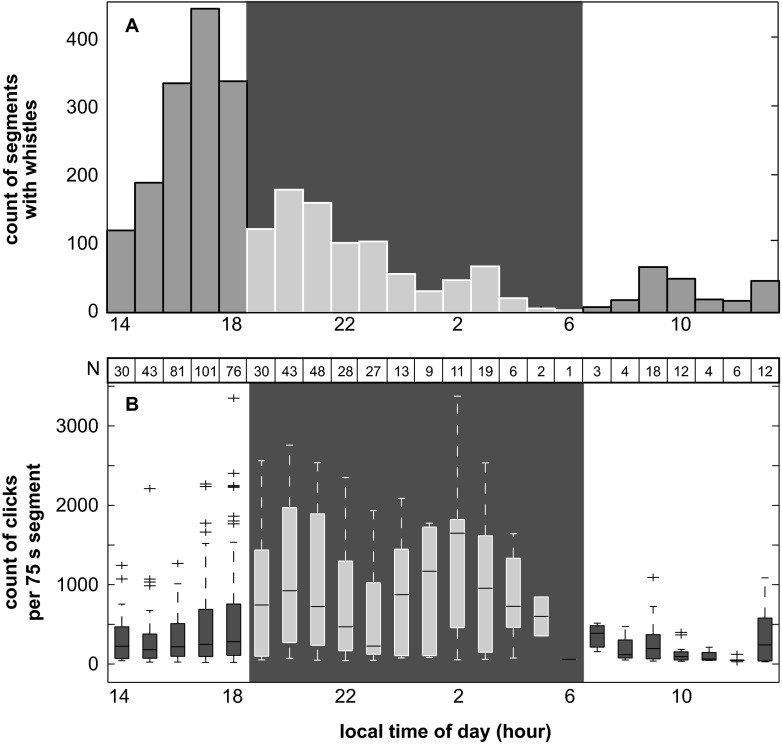
Diel whistle and echolocation click activity of melon-headed whales. **a** Count of 75 s segments with melon-headed whale whistles detected over local time of day showing increased whistling in the late afternoon. **b** Hourly counts of clicks per 75 s segment using reduced number of segments (*N*) over local time of day showing increased echolocation at night. Nighttime is shown with *dark background. Boxplot* shows median hourly counts (*center line*), 1st and 3rd quartile (*solid*). Whiskers (*dashed lines*) denote Tukey inter-quartile outlier test boundaries with outliers marked as *crosses*

Analyst-based study of high-frequency acoustic recording package (HARP) data revealed 176 encounters of melon-headed whale whistle sequences throughout one year of recording. This resulted in 2528 segments of 75-s duration with confirmed melon-headed whale signals. After reducing the dataset to achieve independent samples to one segment every 20 min (duty cycle interval), the analysis was carried out on 627 segments (390 day, 237 night). The automatic click detector found 186,870 echolocation clicks during the day and 243,349 clicks during the night within these segments. Most segments with melon-headed whale whistles were detected in the late afternoon (Fig. 3a). During that period, echolocation click activity was low but increasing towards sunset (Fig. 3b). Fewer segments with melon-headed whale whistles and lower whistling activity in those segments were found during the night. Echolocation click rates were higher throughout the night than during the day (Fig. 3b, Table 1) with a decrease towards sunrise.

**Table 1:** Spectral and temporal characterization of echolocation clicks partitioned by day and night. In each case, characteristics are given for all echolocation clicks, and only those that have peak frequencies (pf) above 20 kHz

		Day all clicks *n* = 390	Night all clicks *n*-237	Day pf >20 kHz *n* = 390	Night pf >20 kHz *n* = 237	Two sample *t*-test with unequal variance (tested on all clicks)		
	Unit	median		median		*t*	d.f.	*p*
Peak frequency	kHz	22.7 (18.3, 29.5)	32.0 (28.5, 39.8)	28.1 (23.4, 39.4)	33.6 (29.7, 40.6)	−20.3	541	<0.0001
Center frequency	kHz	21.7 (18.0, 25.9)	28.2 (21.5, 33.5)	24.7 (20.7, 28.9)	28.8 (24.0, 33.6)	−16.2	392	<0.0001
−3 dB bandwidth	kHz	2.7 (2.3, 5.0)	3.5 (2.6, 5.1)	3.9 (2.7, 6.6)	3.9 (3.1, 5.4)	−6.7	494	<0.0001
−10 dB bandwidth	kHz	16.0 (5.8, 26.1)	19.8 (11.3, 28.0)	22.4 (11.5, 31.2)	22.2 (14.8, 29.2)	−7.0	560	<0.0001
Duration	μs	240 (19, 330)	270 (211, 370)	256 (205, 370)	279 (220, 377)	−4.9	578	<0.0001
Received level (peak-to-peak)	dB re 1 μPa	133 (132, 136)	136 (133, 140)	133 (130, 136)	136 (133, 140)	−11.5	417	<0.0001
Click counts per segment	#	189 (44, 931)	735 (84, 2151)	116 (22, 556)	578 (73, 2028)	−9.8	332	<0.0001

Medians are reported as well as the 10th and 90th percentiles (in parentheses). Student *t*-test provides the significance of diel difference based on segments with all clicks

**Fig. 4:**
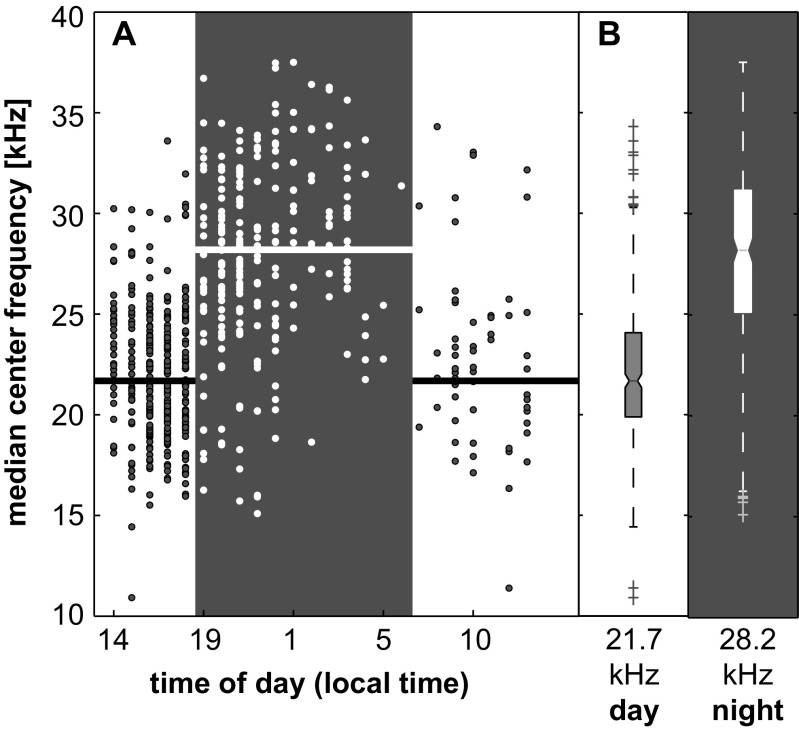
Melon-headed whale echolocation click center frequency versus time of day shows a shift to higher frequency from day to night. **a** Median center frequency of 75 s segments versus time of day. *Horizontal line* indicates day- and nighttime median. **b** *Boxplot* distribution of center frequency shows median (*center line*), 1st and 3rd quartile (*solid*). Whiskers (*dashed lines*) denote Tukey inter-quartile outlier test boundaries with outliers marked as *crosses*. Nighttime is shown with *dark background*

**Fig. 5:**
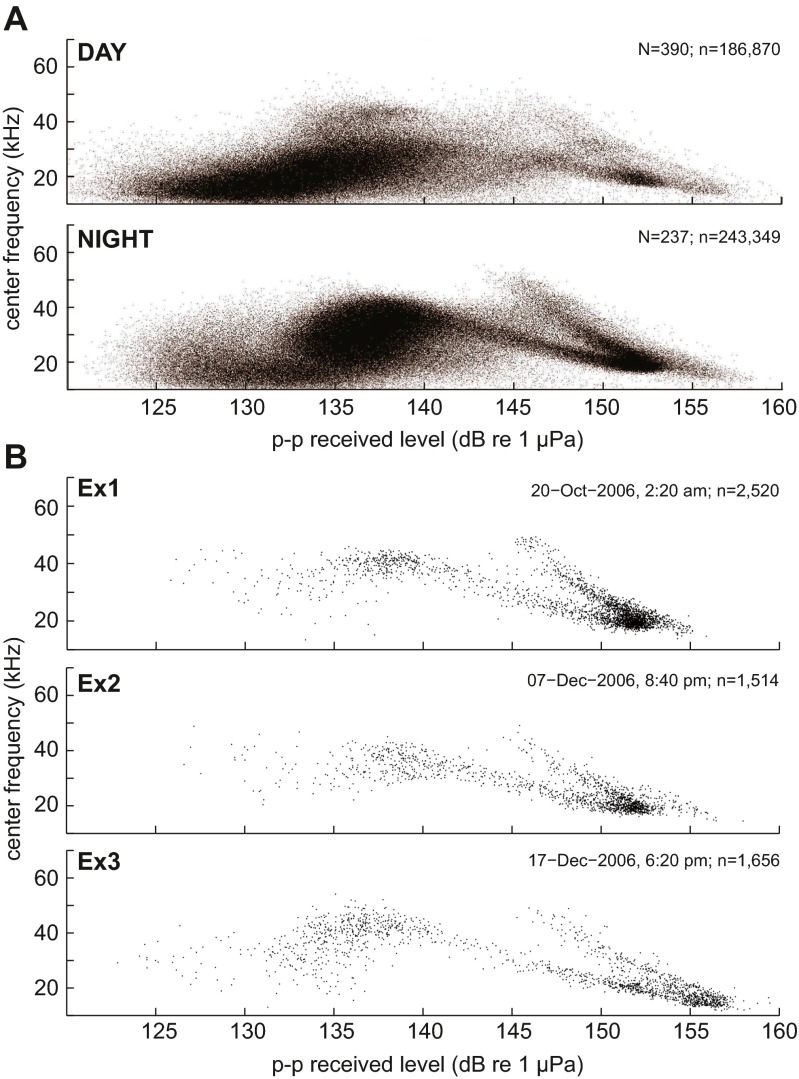
Relationship of center frequency and peak-to-peak click received level showing a distinct shift in echolocation behavior from day to night. This is indicated in an overall shift from lower frequency, lower level clicks during the day to higher frequency and higher amplitude clicks (up to 145 dB re 1 uPa) during the night. When clicks were received above 145 dB, there appeared to be a shift to lower frequencies. **a** All daytime and nighttime segments (*N*) with all clicks (*n*). **b** Three example segments showing this pattern being representative across encounters and not being caused by separate acoustic encounters

**Fig. 6:**
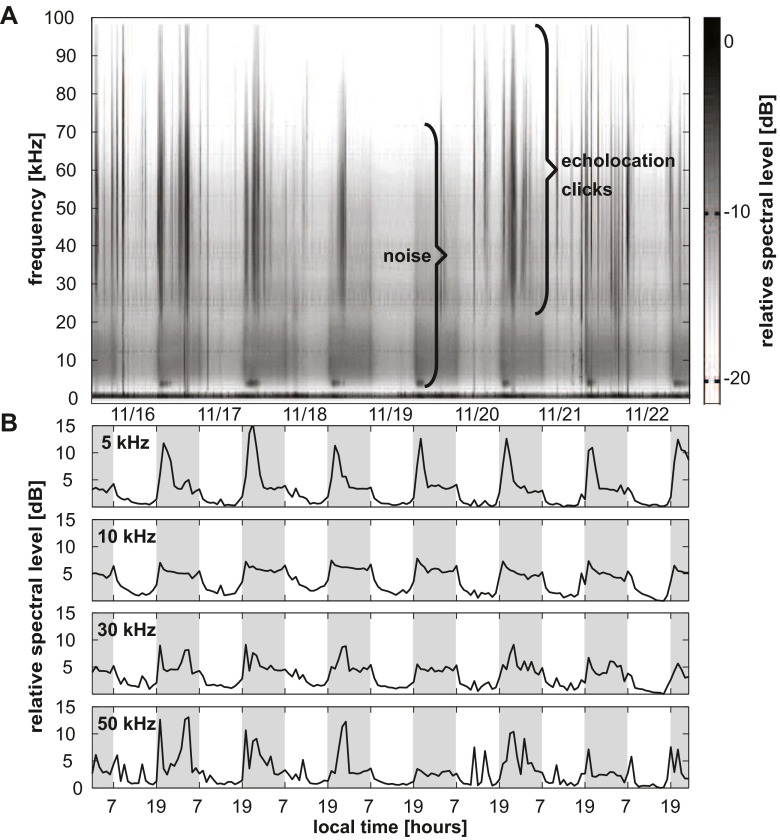
Diel acoustic biotic noise pattern on long-term recording at Palmyra Atoll during 1 week from 16 to 23 November, 2006 starting and ending at midnight local time. **a** Long-term spectral average (LTSA) with frequency versus time (not adjusted for system response). Higher background noise (*broadband medium gray area*) and higher odontocete click activity (*vertical dark lines*) at night. **b** Relative spectral level at 5, 10, 30, and 50 kHz (hourly spectra with 2048-point FFT over 75 s with 0 % overlap, adjusted for system response). Note the especially strong nighttime noise increase at 5 kHz (9 ± 3 dB) between 20:00 and 22:00. Nighttime (19:00–07:00 local time) indicated with *darker background*

**Fig. 7:**
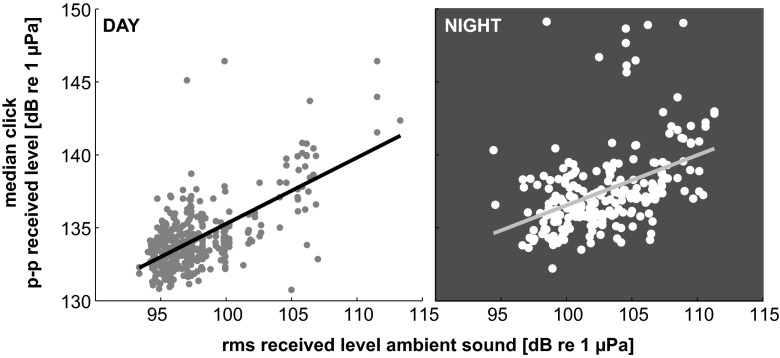
Relationship of median click peak-to-peak received level over 75 s segments and corresponding RMS received level of ambient sound split into day and night. It shows a positive linear relationship (day: *R* ^2^ = 0.43, night: *R* ^2^ = 0.19) with 0.45 and 0.34 dB increase of click received level per dB increase of ambient sound day and night, respectively. Ambient source levels vary as a function of time throughout the day and night, over the course of the lunar cycle, and seasonally based on bio-, anthro-, and geophonic influences

The spectral content of echolocation clicks shifted to overall higher frequencies at night. As a result of this, median peak and center echolocation click frequencies shifted between day and night from 23 to 32 kHz and 22 to 28 kHz, respectively, based on a 0.4-kHz frequency resolution (Fig. 4, Fig. 5, Table 1). With an increase in click frequency occurred an increase of click peak-to-peak received levels. This relationship was stable up to approximately 145 dB re 1 μPa. At higher received levels, center frequency decreased (Fig. 5). Concurrently, a distinct pattern of day and night ambient noise was noted (Fig. 6). This broadband ambient sound pattern appeared to be unique to Palmyra Atoll, and a similar pattern has not been detected at these high levels elsewhere. The detailed origin of the ambient sound was undeterminable, but it resulted in an increase in broadband noise up to about 70 kHz starting at sunset and lasting until sunrise (Fig. 6). Additionally, between about 20:00 and 22:00 local time, there was a distinct recurring noise with a peak at around 5 kHz. RMS received level noise measurements over the full bandwidth resulted in an average 4 dB increase during the night. The increase of peak-to-peak echolocation click received levels was positively related with an increase in ambient sound (Fig. 7) at a rate of 0.45 and 0.34 dB increased click received level per one dB increase in ambient sound day and night, respectively.

## Discussion

Long-term passive acoustic monitoring at Palmyra Atoll revealed diel acoustic behavior of melon-headed whales. Click rates increased in the late afternoon and continued to be high during the night, decreased towards sunrise, and had their lowest values late in the morning and around noon. Echolocation clicks are used by the animals to detect, characterize, and localize a target for spatial orientation or feeding (Au 1993). There is evidence for particularly high rates of echolocation during periods of foraging (e.g., Van Parijs and Corkeron 2001; Nowacek 2005). Detections of segments with melon-headed whale whistles had a strong peak in the afternoon until shortly before sunset, a steep drop-off at the beginning of the night and the lowest detections before sunrise. Whistles have been shown to be important for socializing (Herman and Tavolga 1980; Norris et al. 1994; Janik and Slater 1998; Janik 2000; Lammers et al. 2006). This comparison indicated that whistles and clicks were used selectively during different phases of the day. Variability in hourly detection rates of both whistles and clicks were likely a result of changing animal densities within the detection range of the recorder as well as variability in their behavior.

Melon-headed whales have a diel migratory and behavioral pattern at Palmyra Atoll (Brownell et al. 2009). They rest and socialize along the reef edge near the atoll over shallower water (70 m minimum sighted depth, Fig. 1b) in the morning and early afternoon, with active whistling and low click rates. In the early evening, they move towards deep water to feed on mesopelagic prey (Brownell et al. 2009) and the vocalization pattern reverses with high click rates and less whistling. This is consistent with behavior exhibited by other delphinid species that prey on vertical migrators from the mesopelagic boundary layer during the night (Weilgart and Whitehead 1990; Norris et al. 1994; Nowacek 2005; Benoit-Bird and Au 2009). High rates of nighttime echolocation for melon-headed whales are likely related to diel patterns in prey migration similar to the echolocation behavior of spinner dolphins (Benoit-Bird and Au 2003; Benoit-Bird et al. 2004). Mesopelagic biomass communities undergo a vertical and horizontal migration from deep waters during the day to surface waters at night, with a peak density near the surface around midnight (Enright and Hamner 1967). The diel whistle and click rate pattern of melon-headed whales at Palmyra Atoll supports the hypothesis of nighttime foraging and daytime resting and socializing for this species. Echolocating bats show the most closely related acoustic behavior in the terrestrial environment. They use echolocation at night to forage (Griffin 1958) but also produce social calls under certain circumstances, some of which are related to a diel or seasonal pattern. These social calls are known to occur during mating, to communicate with animals in the roost, to recruit conspecifics, or to defend foraging patches (Bradbury 1977; Rydell 1986; Wilkinson and Wenrick Boughman 1998; Davidson and Wilkinson 2004; Chaverri et al. 2010; Arnold and Wilkinson 2011).

Median values of echolocation click parameters reported here for melon-headed whales are not directly comparable to values derived exclusively from assumed on-axis click measurements. All values were calculated over on- as well as off-axis clicks within a 75-s time segment, and likely, only a small minority of these clicks was on-axis. While on-axis clicks have energy beyond the 100-kHz bandwidth of the HARP, towed array recordings in close vicinity to melon-headed whales with sampling frequency up to 480 kHz show only a 3-kHz increase in median center frequency when grouping more than 50 random on- or off-axis clicks (Baumann-Pickering et al. 2010). This is likely an effect of a dominance of off-axis click in the average spectra and demonstrates its stability across sampling schemes.

Spectral features of melon-headed whale clicks received at the HARP were different from day to night with the entire broadband energy shifted to higher frequency and stronger amplitude at night. The energy started at about 15 kHz during the day and at about 20 kHz during the night. Median center frequency was at 22 kHz during the day at 28 kHz during the night. The higher frequency and stronger amplitude of the recorded clicks could be a result of (1) animals being closer to the HARP and pointing their echolocation beam towards the recorder during nighttime periods of foraging or (2) effort to improve target detectability through an increase of biosonar source level and changes in other click characteristics, such as frequency, are a by-product.

With melon-headed whales moving offshore and to greater depth at night to prey on the mesopelagic boundary layer, one might assume the animals to be closer to the HARP location and more likely to point their echolocation beam downwards towards the hydrophone when possibly diving deeper than during the day. A movement towards the HARP would increase the received level and center frequency due to reduced absorption and geometric spreading loss. Furthermore, directing the echolocation beam downwards during foraging would result in more on-axis clicks, which tend to have a higher received level and higher center frequency.

A number of factors weaken this most parsimonious explanation. Daytime sightings of melon-headed whales were in water depths that would place the animals typically within 300–500 m of the HARPs bathymetry line. Morning sightings were in depths ranging from 70 to 300 m, afternoon sightings in 300–400 m, and one evening sighting in 1300 m. While no night time observations were possible, the tendency towards deeper water as the day moved on coupled with a diet of mesopelagic prey would suggest that the animals were likely offshore of the HARPs’ bathymetry line during the night. The southern reef edge extends over many nautical miles. There is no indication for the probability of echolocating melon-headed whales to be closer to the HARP during the night than during the day.

Furthermore, daytime array recordings (Baumann-Pickering et al. 2010) from groups of up to 1000 animals were made at distances varying from adjacent to the hydrophone to ~2000 m away. The interest of melon-headed whales in these boat and towed array surveys resulted in many strong click trains that were oriented towards the array hydrophone. The median center frequency of these encounters was 31 kHz. The closest the animals were observed to the HARP during a daytime visual encounter was a perpendicular surface distance of about 500 m with a resulting diagonal distance to depth of 800 m. Center frequencies recorded on the HARP during that visual encounter were reported as 30 kHz. Daytime HARP-recorded values presented in this article using the method in Baumann-Pickering et al. (2010), excluding clicks with peak frequencies below 20 kHz, were a median of 29 kHz. This indicates that the change to higher amplitude and higher frequency at night are unlikely a result of the animals being closer to the HARP or pointing their echolocation beam more towards it.

During foraging at night, an echolocation signal with a higher source level should increase prey detection by either increasing the detection range for prey or overcoming, in this particular situation, the increased ambient noise at Palmyra Atoll, or possibly a combination of both. An increased biosonar source level would improve signal detectability based on a higher signal-to-noise ratio. Strong upward frequency shifts together with higher level echolocation clicks were reported for captive beluga and false killer whales in response to noise (Au et al. 1985; Thomas et al. 1988; Thomas and Turl 1990; Turl et al. 1991). Captive bottlenose dolphins are capable of adjusting their echolocation click amplitude yet have more difficulties in precise modification of the frequency structure of their clicks (Moore and Pawloski 1990). The positive relationship of click received levels and ambient noise levels suggests that at least some of the observed amplitude adjustment at night might be a Lombard response (Lombard 1911), and the alteration of other click features, such as higher center frequency, may be a result of the production of higher amplitude clicks.

Bats have been shown to use their echolocation signals in a highly adaptive way depending on the current task and in reaction to changes in the environment. Signal parameters, such as frequency, duration, intensity, and beamwidth, are being adjusted to shape a signal appropriate for the situation (Jakobsen et al. 2013). It is well established that bats adapt the time-frequency structure of their echolocation pulses to the acoustic limits of their habitat and foraging circumstances, which results in the concept of bat guilds (e.g., Neuweiler 1989; Schnitzler et al. 2003; Denzinger and Schnitzler 2013). More recently, levels of intensity and changes in beam width have been investigated. Bats are capable of adjusting their echolocation pulse intensity to overcome frequency dependent attenuation resulting in similar detection ranges for prey across species (Surlykke and Kalko 2008). Additionally, bats are not only adapting the time-frequency components of their signals as they search and approach prey (e.g., Melcón et al. 2007), but they also adjust their signal strength and beamwidth to suit the task (e.g., Koblitz et al. 2011).

## Conclusions

A long-term, collective diel adaptation in free-ranging odontocetes’ biosonar source levels and spectral content either as an adjustment for larger detection distances of prey or to overcome diel changes in ambient sound was discussed. The results show that in the field, just as in captivity, the structure of echolocation clicks is not a rigid entity but changes in response to environmental or situational stimuli.

Future research should test further in the field, how echolocation click structures are modified as a result of increased detection range, as animals change their focal behavior from navigation to foraging, and in the presence of natural or anthropogenic noise. In relation to adjustments due to noise, it seems relevant at what point masking is becoming a disadvantage for equal foraging success and what effect additional energy expenditure due to prolonged foraging has on the ecology of echolocating species.

## Electronic supplementary material

ESM 1 The following supplement accompanies the article in Behavioral Ecology and Sociobiology (2015) (DOCX 75 kb)

## References

[CR1] Aguilar Soto N, Johnson M, Madsen PT, Tyack PL, Bocconcelli A, Fabrizio Borsani J (2006). Does intense ship noise disrupt foraging in deep-diving Cuvier’s beaked whales (*Ziphius cavirostris*)?. Mar Mammal Sci.

[CR2] Amano M, Yoshioka M, Kuramochi T, Mori K (1998). Diurnal feeding by Dall’s porpoise, *Phocoenoides dalli*. Mar Mammal Sci.

[CR3] Arnold B, Wilkinson G (2011). Individual specific contact calls of pallid bats (*Antrozous pallidus*) attract conspecifics at roosting sites. Behav Ecol Sociobiol.

[CR4] Au WWL (1993). The sonar of dolphins.

[CR5] Au WWL, Carder DA, Penner RH, Scronce BL (1985). Demonstration of adaptation in beluga whale echolocation signals. J Acoust Soc Am.

[CR6] Au WWL, Mobley J, Burgess WC, Lammers MO, Nachtigall PE (2000). Seasonal and diurnal trends of chorusing humpback whales wintering in waters off western Maui. Mar Mammal Sci.

[CR7] Baumann-Pickering S, Wiggins SM, Hildebrand JA, Roch MA, Schnitzler H-U (2010). Discriminating features of echolocation clicks of melon-headed whales (*Peponocephala electra*), bottlenose dolphins (*Tursiops truncatus*), and Gray’s spinner dolphins (*Stenella longirostris longirostris*). J Acoust Soc Am.

[CR8] Benoit-Bird KJ, Au WWL (2003). Prey dynamics affect foraging by a pelagic predator (*Stenella longirostris*) over a range of spatial and temporal scales. Behav Ecol Sociobiol.

[CR9] Benoit-Bird KJ, Au WWL (2009). Phonation behavior of cooperatively foraging spinner dolphins. J Acoust Soc Am.

[CR10] Benoit-Bird KJ, Würsig B, McFadden CJ (2004). Dusky dolphin (*Lagenorhynchus obscurus*) foraging in two different habitats: active acoustic detection of dolphins and their prey. Mar Mammal Sci.

[CR11] Bermudez-Cuamatzin E, Rios-Chelen AA, Gil D, Macias Garcia C (2011). Experimental evidence for real-time song frequency shift in response to urban noise in a passerine bird. Biol Lett.

[CR12] Bradbury JW (1977). Lek mating behavior in the hammerheaded bat. Z Tierpsychol.

[CR13] Bradbury JW, Vehrencamp SL (1998). Principles of animal communication.

[CR14] Bridges AS, Dorcas ME (2000). Temporal variation in anuran calling behavior: implications for surveys and monitoring programs. Copeia.

[CR15] Brownell RL, Ralls K, Baumann-Pickering S, Poole MM (2009). Behavior of melon-headed whales, *Peponocephala electra*, near oceanic islands. Mar Mammal Sci.

[CR16] Brumm H (2004). The impact of environmental noise on song amplitude in a territorial bird. J Anim Ecol.

[CR17] Brumm H (2013). Animal communication and noise.

[CR18] Brumm H, Todt D (2002). Noise-dependent song amplitude regulation in a territorial songbird. Anim Behav.

[CR19] Carlström J (2005). Diel variation in echolocation behavior of wild harbor porpoises. Mar Mammal Sci.

[CR20] Chaverri G, Gillam EH, Vonhof MJ (2010). Social calls used by a leaf-roosting bat to signal location. Biol Lett.

[CR21] Cynx J, Lewis R, Tavel B, Tse H (1998). Amplitude regulation of vocalizations in noise by a songbird, *Taeniopygia guttata*. Anim Behav.

[CR22] Davidson SM, Wilkinson GS (2004). Function of male song in the greater white-lined bat, *Saccopteryx bilineata*. Anim Behav.

[CR23] Denzinger A, Schnitzler H-U (2013). Bat guilds, a concept to classify the highly diverse foraging and echolocation behaviors of microchiropteran bats. Front Physiol.

[CR24] Enright JT, Hamner WM (1967). Vertical diurnal migration and endogenous rhythmicity. Science.

[CR25] Farina A (2014). Soundscape ecology: principles, patterns, methods and applications.

[CR26] Feng AS, Narins PM, Xu C-H, Lin W-Y, Yu Z-L, Qiu Q, Xu Z-M, Shen J-X (2006). Ultrasonic communication in frogs. Nature.

[CR27] Foote AD, Nystuen JA (2008). Variation in call pitch among killer whale ecotypes. J Acoust Soc Am.

[CR28] Frankel AS, Yin S (2010). A description of sounds recorded from melon-headed whales (*Peponocephala electra*) off Hawai'i. J Acoust Soc Am.

[CR29] Griffin DR (1958). Listening in the dark: the acoustic orientation of bats and men.

[CR30] Herman LM, Tavolga W, Herman LM (1980). The communications systems of cetaceans. Cetacean behavior: mechanisms and function.

[CR31] Holt MM, Noren DP, Veirs V, Emmons CK, Veirs S (2009). Speaking up: killer whales (*Orcinus orca*) increase their call amplitude in response to vessel noise. J Acoust Soc Am.

[CR32] Holt MM, Noren DP, Emmons CK (2011). Effects of noise levels and call types on the source levels of killer whale calls. J Acoust Soc Am.

[CR33] Jakobsen L, Brinkløv S, Surlykke A (2013). Intensity and directionality of bat echolocation signals. Front Physiol.

[CR34] Janik VM (2000). Whistle batching in wild bottlenose dolphins (*Tursiops truncatus*). Science.

[CR35] Janik VM, Slater PJB (1998). Context-specific use suggests that bottlenose dolphin signature whistles are cohesion calls. Anim Behav.

[CR36] Jefferson TA, Webber MA, Pitman RL (2008). Marine mammals of the world—a comprehensive guide to their identification.

[CR37] Kaiser JF (1990). On a simple algorithm to calculate the “energy” of a signal. IEEE ICASSP, Albuquerque, NM.

[CR38] Kamimura M, Tatsuki S (1993). Diel rhythms of calling behavior and pheromone production of oriental tobacco budworm moth, *Helicoverpa assulta* (Lepidoptera: Noctuidae). J Chem Ecol.

[CR39] Kandia V, Stylianou Y (2006). Detection of sperm whale clicks based on the Teager-Kaiser energy operator. Appl Acoust.

[CR40] Klinowska M (1986). Diurnal rhythms in cetacea: a review. Rep Int Whal Comm (Spec. Issue).

[CR41] Koblitz JC, Stilz P, Pflästerer W, Melcón ML, Schnitzler H-U (2011). Source level reduction and sonar beam aiming in landing big brown bats (*Eptesicus fuscus*). J Acoust Soc Am.

[CR42] Krebs JR, Davies NB (1993). An introduction to behavioral ecology.

[CR43] Lammers MO, Schotten M, Au WWL (2006). The spatial context of free-ranging Hawaiian spinner dolphins (*Stenella longirostris*) producing acoustic signals. J Acoust Soc Am.

[CR44] Lesage V, Barrette C, Kingsley MCS, Sjare B (1999). The effect of vessel noise on the vocal behavior of belugas in the St. Lawrence River Estuary, Canada. Mar Mammal Sci.

[CR45] Lombard E (1911). Le signe de l’élévation de la voix. Ann Mal Oreille Larynx.

[CR46] Lopez PT, Narins PM, Lewis ER, Moore SW (1988). Acoustically induced call modification in the white-lipped frog, *Leptodactylus albilabris*. Anim Behav.

[CR47] Matthews LP, McCordic JA, Parks SE (2014). Remote acoustic monitoring of north Atlantic right whales (*Eubalaena glacialis*) reveals seasonal and diel variations in acoustic behavior. PLoS ONE.

[CR48] Melcón ML, Denzinger A, Schnitzler H-U (2007). Aerial hawking and landing: approach behaviour in Natterer’s bats, *Myotis nattereri* (Kuhl 1818). J Exp Biol.

[CR49] Moore PWB, Pawloski D, Thomas JA, Kastelein RA (1990). Investigation of the control of echolocation pulses in the dolphin (*Tursiops truncatus*). Cetacean sensory systems: field and laboratory evidences.

[CR50] Munger LM, Wiggins SM, Moore SE, Hildebrand JA (2008). North Pacific right whale (*Eubalaena japonica*) seasonal and diel calling patterns from long-term acoustic recordings in the southeastern Bering Sea, 2000–2006. Mar Mammal Sci.

[CR51] Neuweiler G (1989). Foraging ecology and audition in echolocating bats. Trends Ecol Evol.

[CR52] Norris KS, Dohl TP (1980). Behavior of the Hawaiian USA spinner dolphin *Stenella longirostris*. Fish Bull.

[CR53] Norris KS, Wursig B, Wells RS, Wursig M (1994). The Hawaiian spinner dolphin.

[CR54] Nowacek DP (2005). Acoustic ecology of foraging bottlenose dolphins (*Tursiops truncatus*), habitat-specific use of three sound types. Mar Mammal Sci.

[CR55] Oleson EM, Calambokidis J, Burgess WC, McDonald MA, LeDuc CA, Hildebrand JA (2007). Behavioral context of call production by eastern North Pacific blue whales. Mar Ecol Prog Ser.

[CR56] Parks SE, Johnson M, Nowacek D, Tyack PL (2011). Individual right whales call louder in increased environmental noise. Biol Lett.

[CR57] Penna M, Pottstock H, Velasquez N (2005). Effect of natural and synthetic noise on evoked vocal responses in a frog of the temperate austral forest. Anim Behav.

[CR58] Perryman WL, Perrin WF, Würsig B, Thewissen JGM (2009). Melon-headed whale *Peponocephala electra*. Encyclopedia of marine mammals.

[CR59] Potvin DA, Parris KM, Mulder RA (2011). Geographically pervasive effects of urban noise on frequency and syllable rate of songs and calls in silvereyes (*Zosterops lateralis*). Proc R Soc Lond B.

[CR60] Pytte CL, Rusch KM, Ficken MS (2003). Regulation of vocal amplitude by the blue-throated hummingbird, *Lampornis clemenciae*. Anim Behav.

[CR61] Roch MA, Klinck H, Baumann-Pickering S, Mellinger DK, Qui S, Soldevilla MS, Hildebrand JA (2011). Classification of echolocation clicks from odontocetes in the Southern California Bight. J Acoust Soc Am.

[CR62] Rydell J (1986). Feeding territoriality in female Northern bats, *Eptesicus nilssoni*. Ethology.

[CR63] Schaub A, Schnitzler HU (2007). Echolocation behavior of the bat *Vespertilio murinus* reveals the border between the habitat types “edge” and “open space”. Behav Ecol Sociobiol.

[CR64] Scheifele PM, Andrew S, Cooper RA, Darre M, Musiek FE, Max L (2005). Indication of a Lombard vocal response in the St Lawrence River beluga. J Acoust Soc Am.

[CR65] Schmidt U, Joermann G (1986). The influence of acoustical interferences on echolocation in bats. Mammalia.

[CR66] Schnitzler H-U, Moss CF, Denzinger A (2003). From spatial orientation to food acquisition in echolocating bats. Trends Ecol Evol.

[CR67] Sinnott JM, Stebbins WC, Moody DB (1975). Regulation of voice amplitude by the monkey. J Acoust Soc Am.

[CR68] Širović A, Cutter GR, Butler JL, Demer DA (2009). Rockfish sounds and their potential use for population monitoring in the Southern California Bight. ICES J Mar Sci.

[CR69] Soldevilla MS, Henderson EE, Campbell GS, Wiggins SM, Hildebrand JA, Roch MA (2008). Classification of Risso’s and Pacific white-sided dolphins using spectral properties of echolocation clicks. J Acoust Soc Am.

[CR70] Soldevilla MS, Wiggins SM, Hildebrand JA (2010). Spatial and temporal patterns of Risso’s dolphin echolocation in the Southern California Bight. J Acoust Soc Am.

[CR71] Soldevilla MS, Wiggins SM, Hildebrand JA (2010). Spatio-temporal comparison of Pacific white-sided dolphin echolocation click types. Aquat Biol.

[CR72] Staicer C, Spector D, Horn A, Kroodsma D, Miller E (1996). The dawn chorus and other diel patterns in acoustic signaling. Ecology and evolution of acoustic communication in birds.

[CR73] Surlykke A, Kalko EKV (2008). Echolocating bats cry out loud to detect their prey. PLoS ONE.

[CR74] Thomas JA, Turl CW, Thomas JA, Kastelein RA (1990). Echolocation characteristics and range detection by a false killer whale (*Pseudorca crassidens*). Sensory abilities of cetaceans: laboratory and field evidence.

[CR75] Thomas J, Chun N, Au W, Pugh K (1988). Underwater audiogram of a false killer whale (*Pseudorca crassidens*). J Acoust Soc Am.

[CR76] Todd VLG, Pearse WD, Tregenza NC, Lepper PA, Todd IB (2009). Diel echolocation activity of harbour porpoises (*Phocoena phocoena*) around North Sea offshore gas installations. ICES J Mar Sci.

[CR77] Tressler J, Smotherman MS (2009). Context-dependent effects of noise on echolocation pulse characteristics in free-tailed bats. J Comp Physiol A.

[CR78] Turl CW, Skaar DJ, Au WW (1991). The Echolocation ability of the beluga *Delphinapterus leucas* to detect targets in clutter. J Acoust Soc Am.

[CR79] Van Parijs SM, Corkeron PJ (2001). Vocalizations and behaviour of Pacific humpback dolphins *Sousa chinensis*. Ethology.

[CR80] Watkins WA, Daher MA, Samuels A, Gannon DP (1997). Observations of *Peponocephala electra*, the melon-headed whale, in the southeastern Caribbean. Caribb J Sci.

[CR81] Weilgart LS, Whitehead H (1990). Vocalizations of the north Atlantic pilot whale (*Globicephala melas*) as related to behavioral contexts. Behav Ecol Sociobiol.

[CR82] Welch PD (1967). The use of fast Fourier transform for the estimation of power spectra: a method based on a time averaging over short, modified periodograms. IEEE T Acoust Speech.

[CR83] Wieland M, Jones A, Renn SCP (2010). Changing durations of southern resident killer whale (*Orcinus orca*) discrete calls between two periods spanning 28 years. Mar Mammal Sci.

[CR84] Wiggins SM, Hildebrand JA (2007). High-frequency acoustic recording package (HARP) for broad-band, long-term marine mammal monitoring. International Symposium on Underwater Technology 2007 and International Workshop on Scientific Use of Submarine Cables & Related Technologies, IEEE.

[CR85] Wiggins SM, Oleson EM, McDonald MA, Hildebrand JA (2005). Blue whale (*Balaenoptera musculus*) diel call patterns offshore of southern California. Aquat Mamm.

[CR86] Wilkinson GS, Wenrick Boughman J (1998). Social calls coordinate foraging in greater spear-nosed bats. Anim Behav.

[CR87] Würsig B, Würsig M (1979). Behavior and ecology of the dusky dolphin, *Lagenorhynchus obscurus*, in the south Atlantic. Fish B-Noaa.

