# Supplementary material for: Acoustic behavior of melon-headed whales varies on a diel cycle
Source: Behav Ecol Sociobiol. 2015 Jul 25;69(9):1553–63. doi: 10.1007/s00265-015-1967-0 (PMC4534505; doi:10.1007/s00265-015-1967-0)
Supplement: Supplementary file 1 — The following supplement accompanies the article in Behavioral Ecology and Sociobiology (2015) (DOCX 75 kb) [file 265_2015_1967_MOESM1_ESM.docx]

The following supplement accompanies the article in

*Behavioral Ecology and Sociobiology (2015)*

Acoustic behavior of melon-headed whales varies on a diel cycle

**Simone Baumann-Pickering ^1*^, Marie A. Roch ^1,2^, Sean M. Wiggins ^1^, Hans-Ulrich Schnitzler ^3^, John A. Hildebrand ^1^**

1) Scripps Institution of Oceanography, University of California, San Diego, 9500 Gilman Dr. #0205, La Jolla, California, 92093, United States of America

2) Department of Computer Science, San Diego State University, 5500 Campanile Drive, San Diego, California, 92182-7720, United States of America

3) Animal Physiology, Institute for Neurobiology, University of Tübingen, Auf der Morgenstelle 28, 72076 Tübingen, Germany

*E-mail: sbaumann@ucsd.edu

Supplement 1.

Dolphin species discrimination by their whistles at Palmyra Atoll.

This supplement is to document species discrimination using whistles of the most frequently encountered dolphin species at Palmyra Atoll: melon-headed whales (*Peponocephala electra*), bottlenose dolphins (*Tursiops truncates*) and Gray’s spinner dolphins (*Stenella longirostris longirostris*). The discrimination task in the main manuscript was achieved by a trained analyst who was able to exploit whistle shape information. However, even simple descriptive statistics of whistles from different species can provide a reasonably accurate discrimination between melon-headed whale whistles and that of other frequently encountered species.

Results and Discussion

A total of 37 encounters with melon-headed whales (Peponocephala electra), 119 encounters with bottlenose dolphins (Tursiops truncatus), and 19 encounters with Gray’s spinner dolphins (Stenella longirostris longirostris) took place in the waters surrounding Palmyra Atoll during two field seasons. Whistles of all three species were with high significance different from each other in all spectral and temporal aspects (Kruskal-Wallis ANOVA, Chi² test results for minimum frequency: 1587, start frequency: 1848, end frequency: 2339, maximum frequency: 2757, duration: 437). A post-hoc test with Bonferroni correction revealed that every possible pair-wise comparison was significantly different except for whistle duration of bottlenose and spinner dolphins. Melon-headed whale whistles were lowest in all spectral parameters and shortest in duration (Figure 1, Table 2). Their whistle frequency (median) was between a minimum of 9.9 and a maximum of 11.9 kHz while bottlenose dolphin whistles ranged from 11.3 to 16.4 kHz and spinner dolphin whistles from 14.7 to 18.7 kHz.

Testing whistle classification to species level with a two-fold Monte-Carlo test, following a modified bootstrap classification method, showed that the mean error rate for the hypothesis of any group of whistles being produced by melon-headed whales versus one of the other species was 15.9% (median 12.9%, std. dev. ±14.2). The classification test supported that even very simple statistics of whistles are adequate for distinguishing melon-headed whale whistles from those of Gray’s spinner and bottlenose dolphins.

Overall, frequency measures for whistles of all three species appeared to be higher and whistle duration shorter than what has previously been reported for these species in nearby geographic regions (e.g., Oswald et al. 2003; Frankel and Yin 2010). This is most likely an artifact of the annotation procedure. Analyst annotations for whistles were allowed to be fragmented when the signal to noise ratio was such that linking fragments would have needed to be inferred instead of observed. This may have resulted in shorter durations and altered mean and median frequency distributions.

Materials and Methods

Our study area was off-reef of Palmyra Atoll, extending from 162° 15’ W to 161° 57’ W and from 5°57.6’ N to 5°49.2’ N (Fig. 1). Visual and acoustic surveys were conducted from the 26 ft Davis boat, *Zenobia*, during two field seasons from October 16 to November 7, 2006 and September 18 to October 13, 2007 resulting in 26 days of survey effort. During our daytime surveys, a constant visual and acoustic watch was kept with two to three observers and the atoll was circumnavigated choosing a route dependent on sea conditions, mostly within 0.5 to 4 km of the reef edge. The small boat and heavy swell largely precluded use of binoculars for searching. In an average sea state 3 condition, we had a visual detection limitation of about 1 km. When we visually or acoustically detected animals, we approached them for identification, school size estimation, photography and acoustic recordings. Recordings were only made when no other cetacean group was visually detected within a radius of 1 km. During the field encounters, we used a four-channel hydrophone array streamed on 80 m of cable. Depending on the animals’ behavior, the array was either towed with speeds between 2 and 8 knots at a depth of 10-15 m, or deployed as a stationary array with a maximum depth of 80 m. The array was equipped with HS150 hydrophones (Sonar Research & Development Ltd., Beverley, UK), which had a sensitivity of –205 dB re V/μPa and a flat frequency response of ±1 dB over the analysis range. The hydrophones were connected to custom-built preamplifiers and band-pass filter electronic circuit boards similar to those used in High-frequency Acoustic Recording Packages (Wiggins and Hildebrand 2007). Hydrophone signals were digitally sampled with a MOTU Traveler (Mark of the Unicorn, Cambridge, MA) at a sampling frequency of 192 kHz and 16-bit quantization and recorded directly to a computer hard-disk drive with the software *Ishmael* (Mellinger 2001).

To discriminate species by their whistles, a subset of single species array recordings, that contained whistles of melon-headed whales, bottlenose, and spinner dolphins, were analyzed. The selected recordings resulted in an approximately equal recording duration per species, gathered from several sightings to minimize over-representation of individuals (Table 1). Spectrograms were formed from the spectra of 8 ms Hamming-windowed data segments computed every 2 ms. The window length resulted in a 125 Hz frequency bin resolution. Only frequency bins between 5 and 50 kHz, which were within the range for most calls of the species of interest, were retained. Spectrograms were normalized by using a median filter over a 3x3 time-frequency grid followed by a per frequency bin spectral means subtraction over a 5 s window. A trained analyst (SBP) used custom software that permitted the user to interactively specify tonal contours (Roch et al. 2011). The analyst placed points along fundamental frequencies and harmonics through which cubic B-spline curves were fit. B-splines consist of multiple piecewise Bezier curves that are constrained to have smooth transitions between certain points through which the B-spline must pass (Dierckx 1993). In general, the analyst worked on short segments of 3-5 s of recording and would adjust the spectrogram contrast and brightness to most favorably display the tonal contours. Complete tonals as well as fragments were noted. Stepped whistles were recorded as single whistles. When echoes could be clearly distinguished they were not marked. Whistles were distinguished from their harmonics by looking for time-overlapping tonals. Each overlapped pair was noted as being a candidate whistle and candidate harmonic based on their mean frequencies with the lower mean frequency assigned as the candidate whistle. The ratio of the candidate harmonic to whistle frequency was computed across the overlapping section. If the mean ratio was an integer greater than one (within a tolerance of 0.1) and the non-overlapping portion of the candidate harmonic started before or ended after the whistle (tolerance 5% of whistle duration), the candidate harmonic was said to be a harmonic and eliminated from subsequent analysis. In general, this worked well with a small fraction of tonals mislabeled per file. An analysis of a random sample of one file from each species showed that less than 1% of the tonals were mislabeled. After the selection of all whistles, the spectral and temporal characteristics were computed for statistical analysis and species comparison. The whistle parameters were skewed in their distribution and therefore non-parametric statistical tests were calculated. MATLAB was used to compute Kruskal-Wallis one-way analysis of variance tests for species discrimination by whistle parameters. A post-hoc test with Bonferroni correction was run to identify non-significant results among pairs of species.

A two-fold Monte-Carlo test was conducted following a modified bootstrap classification method detailed in Roch et al. (2015). Whistle feature data from each encounter were randomly placed into two partitions. Selection was on an encounter basis to prevent training and testing on different whistles from the same encounter. To increase the variability of the training data, 85% of the samples from the training fold were selected with replacement, and classification was conducted on all whistles in the remaining fold. This was repeated 100 times using 4-mixture Gaussian mixture models as the classifier with one model trained per species for each iteration of the 100 2-fold experiments. Decisions were based on maximum *a-posteriori* joint probability of groups of twenty-three whistles, the smallest number of whistles from any of the encounters.


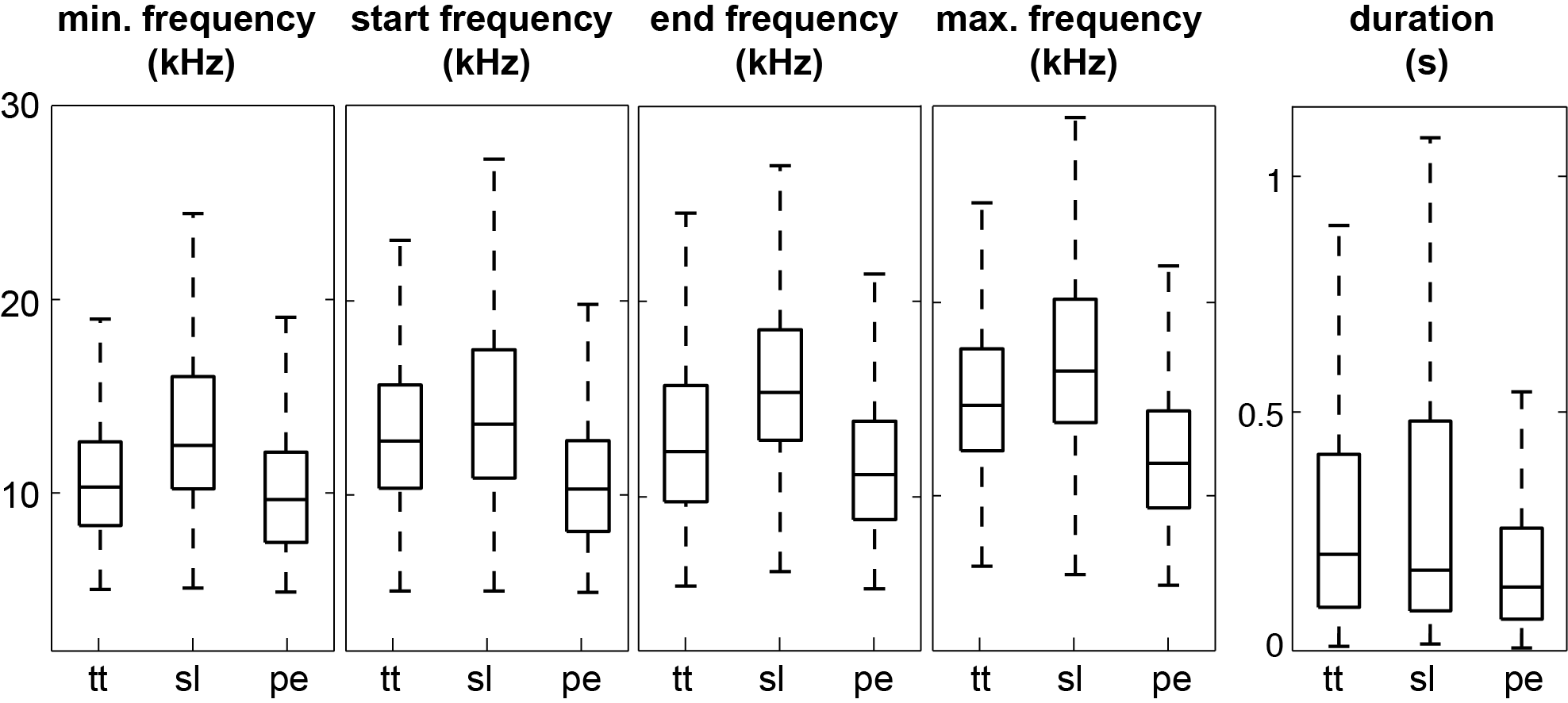


**Fig. S1** Spectral and temporal parameters of whistles of bottlenose dolphins (tt), spinner dolphins (sl), and melon-headed whales (pe)

**Table S1** Data used for whistle comparison of bottlenose dolphin (tt), melon-headed whale (pe) and spinner dolphin (sl) whistles

| ID | duration (m) | # whistles | # animals | date/time (GMT) |
| --- | --- | --- | --- | --- |
| Bottlenose dolphin | | | | |
| tt1 | 1.8 | 221 | 50 | 2006-10-30 23:03:43 |
| tt2 | 16.7 | 1215 | 3 | 2007-09-24 20:53:05 |
| tt3 | 1.9 | 23 | 1 | 2007-10-12 01:06:14 |
| tt4 | 2.6 | 83 | 3 | 2007-10-12 01:20:00 |
| tt-total | 22.9 | 1542 |  |  |
| Melon-headed whale | | | | |
| pe1 | 8.3 | 2491 | 500 | 2006-10-20 20:09:22 |
| pe2 | 7.0 | 1067 | 500 | 2007-09-25 02:30:00 |
| pe3 | 5.3 | 1135 | 500 | 2007-09-27 21:34:44 |
| pe4 | 3.9 | 247 | 500 | 2007-09-28 04:00:00 |
| pe5 | 6.2 | 483 | 700 | 2007-10-04 03:20:00 |
| pe-total | 30.6 | 5423 |  |  |
| Spinner dolphin | | | | |
| sl1 | 2.6 | 179 | 150 | 2006-10-24 22:57:23 |
| sl2 | 7.9 | 814 | 100 | 2006-11-03 21:07:46 |
| sl3 | 10.4 | 921 | 50 | 2007-09-27 22:47:37 |
| sl4 | 5.3 | 1070 | 100 | 2007-10-11 22:56:50 |
| sl-total | 26.1 | 2984 |  |  |

**Table S2** Spectral and temporal whistle parameters for whistles of bottlenose dolphins, melon-headed whales and spinner dolphins. Values are given as mean with standard deviation and median with 10th and 90th percentile in brackets

|  | **bottlenose dolphin** | | **spinner dolphin** | | **melon-headed whale** | |
| --- | --- | --- | --- | --- | --- | --- |
|  | mean | median | mean | median | mean | median |
| minimum frequency [kHz] | 11.1 ±4.5 | 10.3 (6.8 - 16.3) | 13.7 ±5.5 | 12.4 (8.6 - 20.1) | 10.3 ±4.2 | 9.6 (6.0 - 15.5) |
| beginning frequency [kHz] | 13.4 ±4.7 | 12.8 (8.7 - 18.2) | 14.9 ±5.8 | 13.7 (9.1 - 21.9) | 11.1 ±4.4 | 10.3 (6.6 - 16.7) |
| end frequency [kHz] | 12.8 ±4.9 | 12.0 (7.4 - 18.4) | 16.2 ±5.7 | 15.1 (10.6 - 22.6) | 11.7 ±4.7 | 10.9 (6.6 - 17.6) |
| maximum frequency [kHz] | 15.2 ±4.6 | 14.6 (10.5 - 20.0) | 17.4 ±5.7 | 16.4 (11.6 - 24.0) | 12.4 ±4.7 | 11.6 (7.4 - 18.6) |
| Duration [s] | 0.31 ±0.33 | 0.20 (0.05 - 0.73) | 0.33 ±0.36 | 0.17 (0.05 - 0.90) | 0.19 ±0.18 | 0.13 (0.03 - 0.42) |

**References:**

Dierckx P (1993) Curve and Surface Fitting with Splines. Oxford Science Publications, Oxford

Frankel AS, Yin S (2010) A description of sounds recorded from melon-headed whales (Peponocephala electra) off Hawai'i. J Acoust Soc Am 127:3248-3255

Mellinger DK (2001) Ishmael 1.0 user’s guide. NOAA Technical Report No OAR-PMEL-120 Seattle: NOAA Pacific Marine Environmental Laboratory

Oswald JN, Barlow J, Norris TF (2003) Acoustic identification of nine delphinid species in the eastern tropical Pacific Ocean. Mar Mammal Sci 19:20-37

Roch MA, Brandes TS, Patel B, Barkley Y, Baumann-Pickering S, Soldevilla MS (2011) Automated extraction of odontocete whistle contours. J Acoust Soc Am 130:2212-23

Roch MA, Stinner-Sloan J, Baumann-Pickering S, Wiggins SM (2015) Compensating for the effects of site and equipment variation on delphinid species identification from their echolocation clicks. J Acous Soc Am 137:22

Wiggins SM, Hildebrand JA (2007) High-frequency Acoustic Recording Package (HARP) for broad-band, long-term marine mammal monitoring. International Symposium on Underwater Technology 2007 and International Workshop on Scientific Use of Submarine Cables & Related Technologies, IEEE:551–557
